# Polymer and Composite Materials in Two-Phase Passive Thermal Management Systems: A Review

**DOI:** 10.3390/ma16030893

**Published:** 2023-01-17

**Authors:** Ali Ahmed Alqahtani, Volfango Bertola

**Affiliations:** Laboratory of Technical Physics, School of Engineering, University of Liverpool, Brownlow Hill, Liverpool L69 3GH, UK

**Keywords:** polymer materials, composite materials, heat transfer, two-phase flow, heat pipes, thermosyphons, capillary loops, pulsating heat pipes

## Abstract

The application of polymeric and composite materials in two-phase passive heat transfer devices is reviewed critically, with a focus on advantages and disadvantages of these materials in thermal management systems. Recent technology developments led to an increase of the power density in several applications including portable electronics, space and deployable systems, etc., which require high-performance and compact thermal management systems. In this context, passive two-phase systems are the most promising heat transfer devices to dissipate large heat fluxes without external power supply. Usually, heat transfer systems are built with metals due to their excellent thermal properties. However, there is an increasing interest in replacing metallic materials with polymers and composites that can offer cost-effectiveness, light weight and high mechanical flexibility. The present work reviews state-of the-art applications of polymers and composites in two-phase passive thermal management systems, with an analysis of their limitations and technical challenges.

## 1. Introduction

Thermal management systems play a vital but often overlooked role in several high-tech systems and devices, ranging from small portable electronic devices to large data centeres [1,2,3,4,5,6], since they ensure the temperature requirements of such systems, and of their components, are met, which in turn enables optimal working conditions of devices and significantly extends their lifetime. The increasing technology challenges posed by volumetric density scaling in integrated, functional, or packaged systems [7] drive the interest in high-performance, compact heat transfer devices that can efficiently manage large heat fluxes. In this context, passive two-phase thermal management systems such as heat pipes represent a very promising, simple and cost effective technology compared to other heat transport devices [8].

Two-phase passive thermal management systems are heat transfer devices where a process of liquid–vapour circulation is established between an evaporator and a condenser by exploiting both sensible heat and latent heat in the heat transfer process. They are highly thermal conductors of which their thermal conductivities are many times greater than thermal conductivities of solid materials (e.g., pure copper). Therefore, they have been widely used as thermal control systems for several advanced applications [9].

Similar to other thermal management systems, passive heat transfer devices are usually built with metallic materials, often with high thermal conductivity such as copper. However, recent advances in technology such as foldable and flexible electronic components and devices, soft robotics, as well as spacecraft components, often have additional requirements of mechanical flexibility, low-cost, and/or low weight, which sometimes are difficult to achieve using metallic materials. Thus, there is a growing interest in replacing metals in full or in part with polymer and/or composite materials, which offer cost-effectiveness, light weight, high mechanical flexibility, resistance to corrosion and ease of manufacturing, at the price of a generally much lower thermal conductivity.

This paper presents a review of the existing applications of polymer and composite materials in two-phase passive thermal management systems. Firstly, an overview of the material properties relevant to heat transfer devices is presented. Secondly, a survey of state-of-the art two-phase passive heat transfer devices fabricated using polymer or composite materials, with focus on natural circulation, gravity-driven heat transfer devices (thermosyphons) and on capillary-driven systems (capillary loops, conventional and pulsating heat pipes). Finally, the main limitations and technical challenges of polymer and composite materials relative to their use in thermal management systems are discussed, with an outlook towards potential new research trends.

## 2. Polymer and Composite Materials

**Figure 1:**
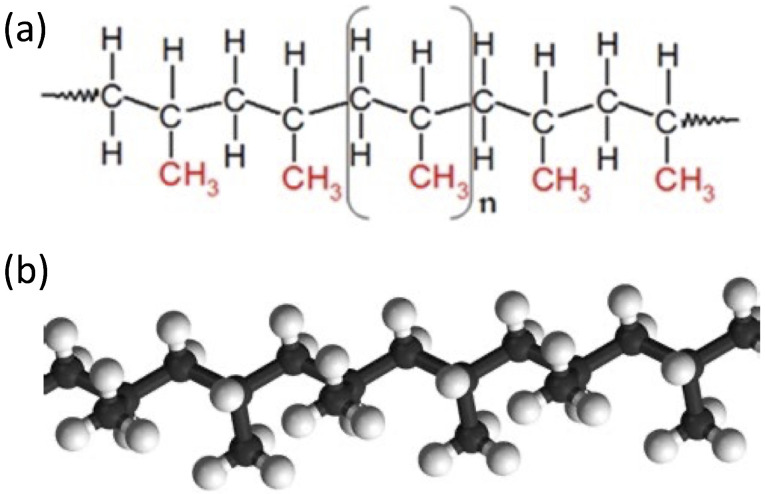
Structure of polypropylene molecular chain (**a**) and its three-dimensional graphical reconstruction (**b**).

Polymer materials are constituted by high molecular weight molecules, which are composed of a large number of units called monomers, connected by covalent bonds, resulting in high molecular weights that can exceed several millions [10]. A common example is polypropylene ${\left(C_{3}H_{6}\right)}_{n}$, where the basic propylene unit is repeated *n* times to form a long linear chain, as shown in Figure 1. Polymers can be found in nature (e.g., natural rubber, starch, cellulose, DNA, etc.); however, those that have the greatest importance in industry are produced by chemical synthesis, usually through catalytic processes [11]. The use of polymeric materials is widespread, and often enables the effective replacement of other materials such as metals, timber and natural fibers. This is largely due to a combination of features such as good mechanical strength, resistance to corrosion and to several chemical agents, electrical and thermal insulation properties, low weight, cost-effectiveness, and ease of manufacturing [12,13,14].

The most distinctive characteristic of polymeric materials as compared with metallic materials, which generally exhibit an elastic response to an applied stress or deformation, is their visco-elastic behaviour [15]. In particular, the response of polymeric materials to an applied stress or deformation is time-dependent, and results in creep, i.e., the time-dependent deformation resulting from a constant applied stress, and into stress relaxation, i.e., the exponentially decaying stress resulting from a constant applied deformation. From the microscopic point of view, this behaviour is usually related to conformational rearrangements of the macromolecules which compose the polymer in order to attain the state of maximum conformational entropy [10]. Other differences of polymeric materials with respect to metals are significantly lower thermal and electrical conductivities, poor surface wettability, and permeability towards non-condensable gases and moisture [16,17]. In addition, several polymeric materials are subject to aging and/or degradation, especially when exposed to sunlight, moisture and heat [18]. These characteristics often make it difficult to use these materials in heat transfer applications [14,16].

Most polymeric materials can be sorted into one of the following three categories: thermoplastics, thermosetting materials, elastomers. The names of the most commonly used polymers belonging to each of these categories are listed in Table 1. Thermoplastics are plastic polymer materials consisting of linear or branched chain molecules with weak intermolecular bonds and strong intramolecular bonds, that become highly deformable at a certain temperature and solidify upon cooling. Thus, they can be shaped/reshaped several times, and are typically used to produce parts by different polymer processing techniques such as extrusion and injection molding. Unlike thermoplastics, thermosetting polymers form strong, irreversible intermolecular bonds when heated, therefore they cannot be reshaped or recycled once solidified. Elastomers are very flexible amorphous materials characterized by weak intermolecular forces, low Young’s modulus and high failure strain [19]. Thus, they can undergo very large deformation under a given force, and recover their original shape once the force is removed. An additional category of linear chain polymers is that of fibers, where molecules are bundled together by hydrogen bonds or strong dipole–dipole attraction, which results in a very high tensile strength and negligible elasticity. Polymer fibers are often of natural origin, such as cellulose, wool, silk, etc.; however, to date, they have not found significant applications in the fabrication of heat transfer devices. A common feature to most polymer materials is the gradual and reversible transition from a viscoelastic or rubbery state to a hard and relatively brittle state upon cooling, which is usually referred to as a glass transition. In particular, for each polymer one can identify a glass-transition temperature, which indicates the range of temperatures over which this glass transition occurs.

To improve certain properties of polymeric materials (e.g., mechanical properties, thermal conductivity, etc.), they are often composited with different polymers and/or other materials (metals, natural fibres, etc.). This results in a class of composite or engineered materials which often exhibit exceptional properties in terms of tensile, compressive, flexural and impact strength, Young’s modulus, thermal expansion coefficient, corrosion resistance, and fatigue resistance [20].

In many cases, composite materials consist of two or more materials, one of which (usually a polymer) acts as a continuous matrix and the others are dispersed fillers, selected according to criteria depending on the application. In thermal management applications, high thermal conductivity fillers are often chosen to enhance the conduction heat transfer rate of the material. To date, several types of high thermally conductive fillers are being used, such as metallic, carbon, and ceramic fillers [21,22]. Metallic fillers (e.g., nickel, copper, aluminum and silver) and carbon fillers (e.g., graphite, graphene, carbon nanotube and carbon fibers) are quite effective and can improve significantly the thermal conductivity of polymers [23,24,25,26,27,28,29,30,31,32,33]. However, they can also lead to an increase in electrical conductivity, which might limit the range of their applications [2,34]. Ceramic fillers (e.g., alumina, silica, aluminum nitride, boron nitride, silicon nitride and silicon carbide) could also improve thermal conductivity without reducing electrical insulation properties and, in addition, they can act as a barrier towards incondensable gases and moisture [35,36,37,38,39,40]. Other factors that determine the mechanical and thermal properties of the composite material are, in addition to the matrix and filler properties, the amount of filler, its size, shape, spatial arrangement/orientation, and the interaction and adhesion between the matrix and the filler. Thus, several parameters should be taken into account when designing and processing composite materials to meet the requirements of thermal management applications.

Polymeric composites can be processed with either thermoplastic or thermosetting polymers using a range of advanced manufacturing techniques, such as high-pressure injection, stamping, hot compression molding, resin injection, low-temperature and pressure compression molding, centrifugal molding, pultrusion, continuous impregnation, filament and tape winding, hand layup, spray layup, autoclaving, extrusion, additive manufacturing and many more [20,41,42,43]. The manufacturing technique is determined based upon various factors such as the material compatibility, the processing parameters, the size, geometry, volume of production, functionality and cost of the final product.

## 3. Polymer and Composite Materials in Two-Phase Passive Thermal Management Systems

Two phase passive thermal management systems are heat transfer devices which can transfer heat from one point to another thanks to the thermally-induced circulation and/or oscillation of a heat transfer fluid without using any external forces, due to the interplay effects of three physical phenomena: phase change, capillarity and gravity. There are several types of two-phase passive heat transfer devices which can be distinguished based on the peculiar geometry and working principle: the conventional heat pipe, the loop heat pipe and the capillary-pumped loop heat pipe, the thermosyphon, and the pulsating heat pipe [9,44,45]. Whilst the early prototypes were built with metals, the use of polymers material to replace metallic parts, partially or entirely, is often desirable in order to reduce cost and weight, and to obtain mechanical flexibility. A comprehensive review on such endeavours is discussed below to provide an overview on the use of polymers in the context of state-of-the-art two-phase passive heat transfer technologies.

### 3.1. Thermosyphons

**Figure 2:**
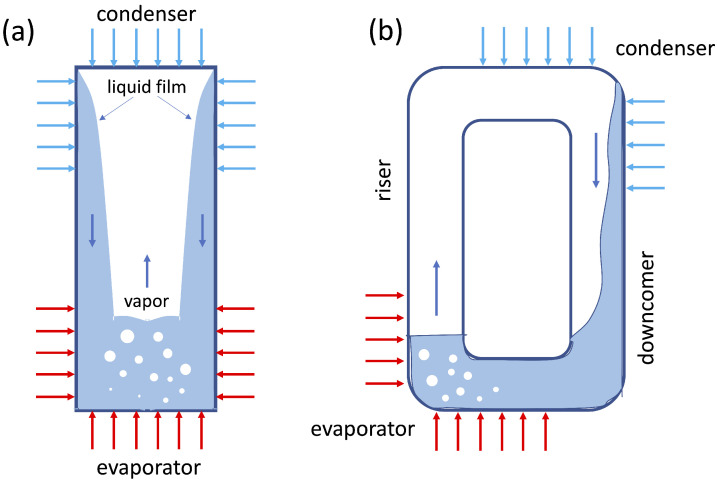
Schematic of single thermosyphon pipe (**a**) and loop thermosyphon pipe (**b**) [56].

A thermosyphon (TS) is a passive heat transfer device which exploits the buoyancy induced by a temperature gradient to circulate a heat transfer fluid, either in an open or in a closed loop. This concept, which has been known for centuries, equally applies to single-phase and two-phase flows; however, the latter can usually achieve better performances by exploiting the phase transition (latent) heat. Since the fluid circulation is due to buoyancy, these devices require gravity assistance, and their performance strongly depends on their spatial orientation. Modern thermosyphons can be dated back to the 1800s [46], although similar systems appeared in the first century A.D. [47]. They consist of a vacuumed closed tube partially filled with heat transfer fluid, and composed with three main sections: the evaporator, the adiabatic section and the condenser, as shown in Figure 2. Thermosyphons always operate in bottom heating mode, i.e., with the evaporator always placed below the condenser to allow the return of condensation to the heat source under the effect of gravity [48]. The main applications of thermosyphons applications are, for example, electronics, energy storage systems, solar systems, waste heat recovery, and nuclear systems [49,50,51,52,53,54,55].

**Figure 3:**
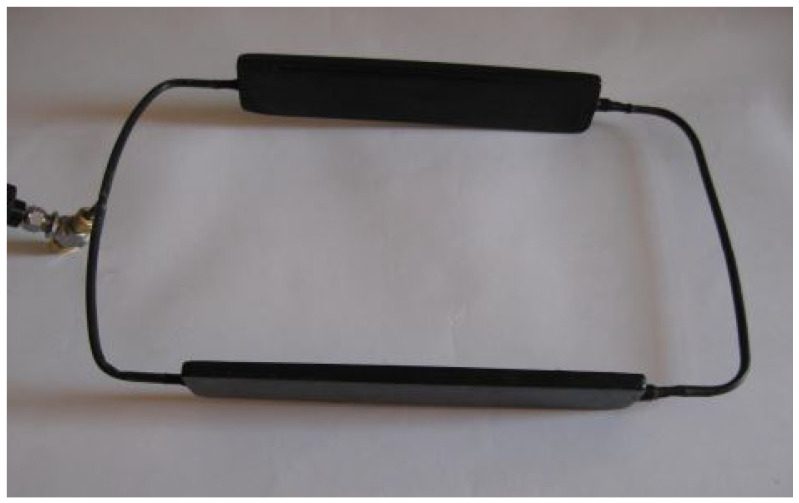
Flat loop thermosyphon built with a polyamide polymer composite [62].

In the past few decades, several conceptual and experimental studies have been conducted on thermosyphons [46,56,57,58]. However, very few of these studies considered polymer-based TSs or partially used polymer within the TS structure. Gernert and Donovan [59] manufactured and tested a proof-of-concept polymeric thermosyphon to be integrated into the radiator system of a lunar base, built with a laminated material consisting of polypropylene and aluminium films. Although the use of the composite polymer enables a significant weight reduction, and allows a compact storage of the device during transportation, the thermal performance is strongly affected by the large temperature excursions experienced in space. Sukchana and Pratinthong [60,61] fabricated and tested two-phase closed loop thermosyphons partially built with polymer. In particular, PTFE tubes were used for the adiabatic section, while copper tubes were used for the evaporator and the condenser, respectively. The device exhibited good thermal performance for different design and operation parameters; however, bending and tilting the TS decreased the thermal performance compared to the vertical orientation without bending. Grakovich et al. [62] developed a polymer loop thermosyphon featuring flat-plate evaporator and condenser connected by flexible transport lines, as shown in Figure 3. Both the condenser and the evaporator casings were built with a polyamide composite containing nano carbon filaments and nano particles to increase their effective thermal conductivity, while the internal surfaces were covered with rectangular capillary grooves to enhance heat transfer. Pure polyamide with smooth surfaces was used for the adiabatic transport lines, where no heat transfer occurs. The device showed high heat transfer performance and ensured gas tightness over extended periods of time [62,63]. As a final remark, several authors used polymer materials (especially polycarbonate and nylon) in laboratory prototypes of thermosyphons to create viewports for flow visualization [64,65,66,67,68].

### 3.2. Conventional Heat Pipes

**Figure 4:**
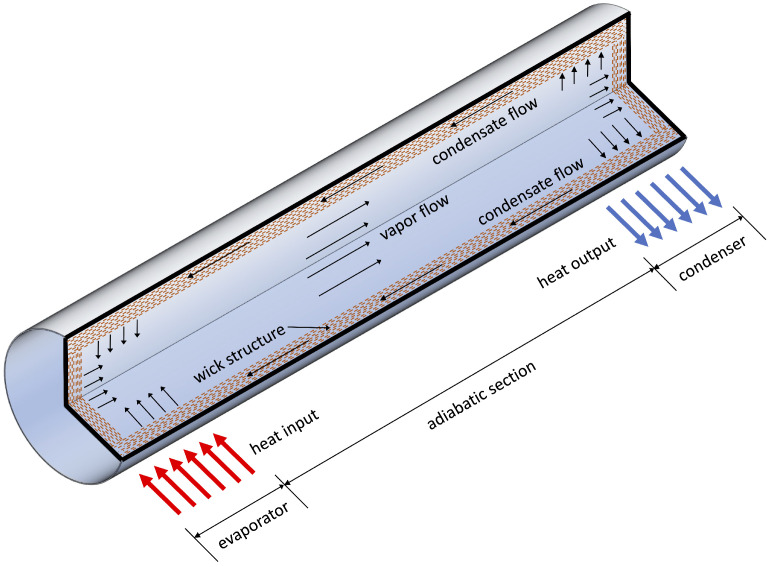
Schematic of conventional heat pipe.

Conventional heat pipes (HPs) are a common type of two phase passive heat transfer device which can achieve large heat transfer rates over relatively long distances and under relatively small temperature differences between the heat source and the heat sink. The concept of HP was first introduced by Gaugler in 1944s [69], and was further developed and built by Grover and co-workers in 1963s [70]. Conventional HPs typically consist of a sealed tube having the inner surface coated with a wick layer, and partially filled with a heat transfer fluid. The HP tube can be divided into three main sections; the evaporator, which is the part in contact with the heat source, the condenser, which is in contact with the heat sink, and the adiabatic section, which connects the evaporator with the condenser, as shown in Figure 4. Upon sufficient heating, the heat transfer fluid evaporates and expands through the adiabatic section until it reaches the condenser, where it releases the phase transition heat and returns to the liquid phase. The condensate flows back to the evaporator through the porous structure of the wick due to capillarity. Depending on the HP orientation, the fluid circulation may be assisted by gravity. This process is self-sustained as long as a sufficient temperature difference is maintained between the evaporator and the condenser. Heat pipes typically have a very high effective thermal conductivity, while the absence of moving parts makes them extremely reliable [71]. Therefore, HPs are widely used in many applications such as power generation, heating, ventilation, air conditioning, the aerospace industry, mobile devices, computers, RF systems, data centres, high-power LEDs, solar cells, and solid-state laser light sources [6,72,73,74,75]

Since they were introduced, heat pipes have been manufactured according to different designs, using mainly metallic materials such as copper and aluminium [6,73,74,75,76]. However, the use of polymer materials in heat pipes was much less frequent. Wessel and Tom [77] developed and tested a flat miniature heat pipe, enbedded in the laminated structure of a printed circuit board (PCB), which consists of a multilayer structure of polymer layers alternating with bonding layers. The HP inner surfaces were copper plated to improve thermal conductivity and prevent gas permeation through the wall, while arrays of thin copper cylinders (thermal vias) were used to enhance heat transfer from the HP to the PCB surface in the evaporator and in the condenser sections. The wick structure consisted of axially oriented capillary microgrooves engraved in the copper layer. The HP exhibited a high equivalent thermal conductivity of around 2939 W/mK, which is seven times higher than the thermal conductivity of pure copper.

Oshman et al. [78] developed a polymeric flat-plate HP where the casing was built with liquid-crystal polymer (LCP) films featuring copper thermal vias in the non-adiabatic sections, while the wick structure consisted of a copper micropillar/woven mesh, similar to other works [79,80]. The thermal performance was assessed by evaluating the thermal conductivity, which reached values up to 830 W/mK, two times higher than pure copper. The same authors [81] also designed and manufactured a lightweight and flexible flat-plate HP using a composite polymer material, consisting of laminated sheets of low-density polyethylene terephthalate (LDPET), aluminium, and polyethylene, with a wick structure consisting of a triple-layer sintered copper woven mesh. The device thermal conductivity was found to be about 4.6 times the conductivity of the copper reference. A similar approach was proposed in [82], where flexible polyethylene-terephthalate (PET) plastic films and a copper mesh supported by rubber pillars were used for the HP casing and the wick structure, respectively. Another attempt to use laminate sheet but with polyamide multi-layer laminate was reported in [83].

Hsieh and Yang [84] fabricated a flexible silicon rubber hybrid flat-plate HP with copper thermal vias in the condenser and evaporator sections. The wick structure was made of two layers of woven copper mesh embedded in the pipe internal surface. Results confirmed that the polymer casing can replace copper as the heat transfer performance is almost comparable. Lewis et al. [85] built a fully polymeric heat pipe using polyimide as a casing material and lithography-defined micropillars as a wick structure. The effective thermal conductivity of the device was 541 W/mK, which is about 10% higher than the copper reference.

Yang et al. [86] produced a flat polymer heat pipe for the thermal management of electronics made of FR4, a composite polymer material commonly used in the printed circuit board (PCB) industry as a substrate. In particular, the HP consists of a copper sheet sandwiched between two FR4 sheets, while the wick structure consists of copper mesh, and thermal vias in the evaporator and the condenser. Their results show the polymeric heat pipe can work without gravity assistance, and thermal vias significantly decrease the equivalent thermal resistance, $R=\left(T_{ev}-T_{cond}\right)/\dot{Q}$, where $T_{ev}$ and $T_{cond}$ are the evaporator and condenser average temperatures, respectively, and $\dot{Q}$ is the heat input.

Chao et al. [87] developed a heat pipe using a fluororubber tube for the adiabatic section, and a copper tube for both the evaporator and the condenser, with copper mesh as the wick structure. The HP was tested under a wide range of experimental parameters, and was successfully used as a thermal control system in foldable electronics.

**Figure 5:**
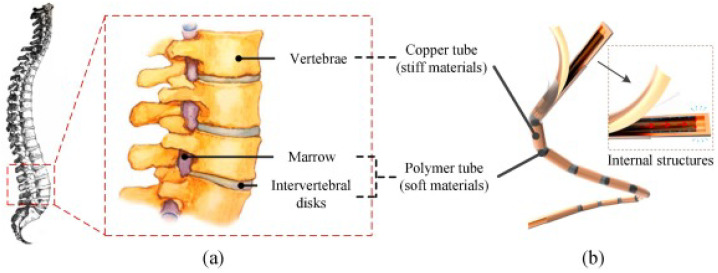
Schematic of human spine (**a**) and a bionic flexible heat pipe (**b**) [90].

Yang et al. [88] developed a flexible heat pipe with bio-inspired wick structures, integrating a polyurethane polymer connector between copper condenser and evaporator. In particular, the wick structure consisted of a bio-inspired superhydrophilic strong-base-oxidized copper mesh with multi-scale micro/nano-structures [89]. The heat transfer performance was evaluated for several experimental parameters, resulting in a minimum thermal resistance of 0.008 K/W. Very recently, an innovative multi-stage flexible heat pipe inspired by the structure of the human spine was developed [90], as shown in Figure 5. The HP design consisted of several stiff copper tubes inserted into a continuous flexible heat-shrinkable sleeve, separated by two layers of polyvinyl chloride (PVC) sealing tapes, and a hydrophilic copper mesh as a wick structure inserted into the tube. The HP demonstrated high performance in all orientations.

### 3.3. Loop Heat Pipes/Capillary Pumped Loops

**Figure 6:**
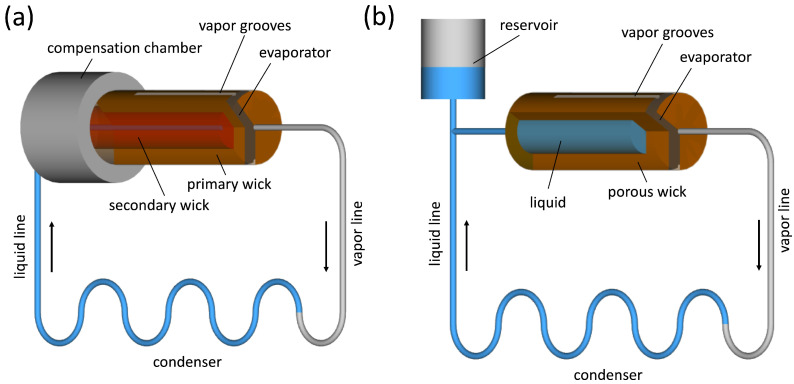
Schematic of a loop heat pipe (**a**), and a capillary-pumped loop heat pipe (**b**) [91].

Loop heat pipes (LHP) and capillary pumped loops (CPL), schematically displayed in Figure 6, are advanced types of conventional heat pipes where the amount of wick structure distributed along the pipe is significantly smaller in order to reduce pressure losses [9,44]. Although both systems are based on a similar working principle, the LHP development dates back to the former Soviet Union, while the CPL concept was initially proposed in the United States in 1966 [45]. In both systems, the wick structure is located in the evaporator section only, while the rest of the system consists of a tube with smooth inner wall, which enables the transfer of heat over long distances with minimal pressure loss as compared to conventional heat pipes. The main difference between LHPs and CPLs is that CPLs have a separated temperature-controlled reservoir to control the operating temperature, while LHP has reservoir (also called compensation chamber) built within the evaporator so that temperature cannot be controlled.

LHP and CPL are efficient systems and are suitable for several applications, including aerospace, cryogenics, solar systems and electronics [92,93,94,95,96,97,98,99]. However, some of these applications require high performance and/or high mechanical flexibility of such systems [100,101,102], which resulted in some attempts to develop LHP or CPL using polymer materials. In addition, the heat leakage from the evaporator to the compensation chamber, a well-known issue which affects adversely the performance of LHPs [103,104], can be mitigated using polymer wicks. Gerner and Brown [105] developed a flexible loop heat pipe using stainless steel, copper and a porous polymer as a wick structure in the evaporator. Similar attempts with LHPs or CPLs using PTFE (Polytetrafluoroethylene), UHMW-PE (ultra-high-molecular-weight polyethylene), PP (polypropylene) and PE (polyethlene) wicks were conducted in [100,102,106,107,108,109,110,111,112,113,114,115]. Ye et al. [116] developed a polymer-based miniature loop heat pipe with a silicon substrate as a cooling system for high brightness light-emitting diodes (LEDs). The device was built by assembling acrylic polymer and silicon rubber tubes. Results confirmed that such device can be beneficial for future LEDs as it contributes well to their thermal management. Phan and Nagano [117] fabricated and tested LHP using polydimethylsiloxane (PDMS) and glass, with a PTFE wick. The device operated well under different working conditions with maximum heat transfer capacity of 9W. Very recently, two miniature LHPs with novel hybrid structures of evaporators were developed and investigated in [118]. The materials used were PDMS and glass, and PDMS and stainless steel, respectively. Both LHPs have silicon transport pipes and PTFE wick structures, and exhibited good performance with heat transfer rates up to 14 W. Finally, other attempts to use polymer materials (perfluoroalkoxy alkanes and polycarbonate) as a part of the tube connections or the structure casing for visualization purposes, are reported among others in [115,119,120,121,122].

### 3.4. Pulsating Heat Pipes

**Figure 7:**
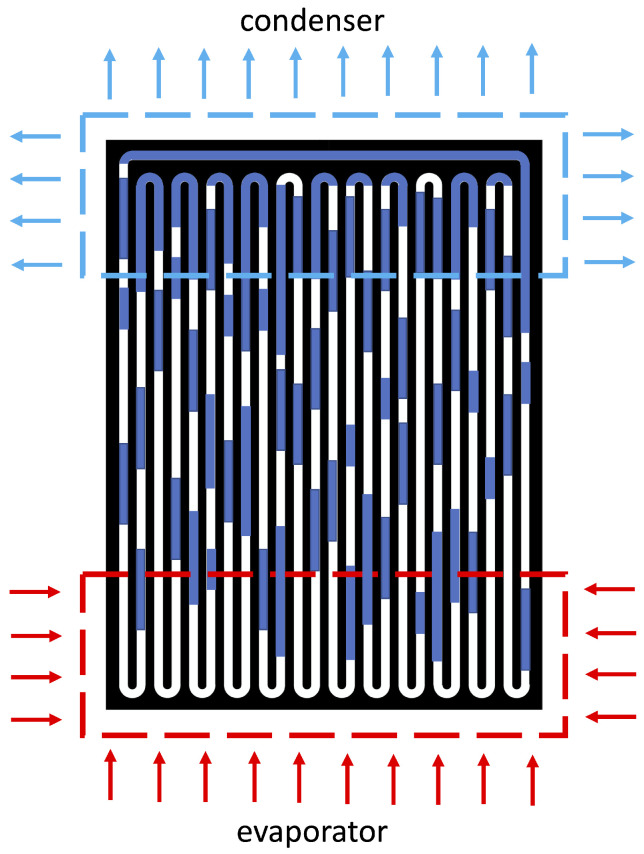
Schematic of a pulsating heat pipe.

Pulsating heat pipes (PHPs), also known as oscillating heat pipes (OHPs), initially proposed by Akachi in the 1990s [123], are the latest evolution among two-phase passive heat transfer technologies. Pulsating heat pipes are closed loop two-phase passive heat transfer devices, operating in thermal self-driven mode under the effect of capillarity, phase change and pressure difference [124,125]. They consist of a vacuumed capillary loop bent in several turns passing through the evaporator and the condenser, and partially charged with heat transfer fluid at the saturation condition, as shown in Figure 7. Upon heating the PHP in the evaporator section, pressure instabilities between the heat source and sink along with capillarity effect induce complex two-phase flow as a train of liquid slugs and vapor plugs.

The PHP has many advantages with respect to the other two phase passive devices, such as: a completely passive operation without the need of a wick structure, structure simplicity and compactness, ease of manufacturing, and also the ability to operate against gravity [45]. Because of these unique features, PHPs can be integrated within several applications including aerospace, solar systems, heat recovery systems, electronics, fuel cells and batteries, and cryogenics [126,127,128], although further applications are being explored [128,129]. The use of plastics instead of metallic materials would be desirable in many applications where mechanical flexibility is required in addition to low-weight and low-cost constraints, at different power and size scales. These include portable and foldable electronics such as foldable smartphones, flexible RFID and OLED systems (micro-scale), soft robotics, e-textiles (medium scale), electric vehicle battery packs (macro-scale). Other relevant applications are in low-temperature heating of surfaces, such as in the case of de-icing, and distributed energy harvesting.

**Figure 8:**
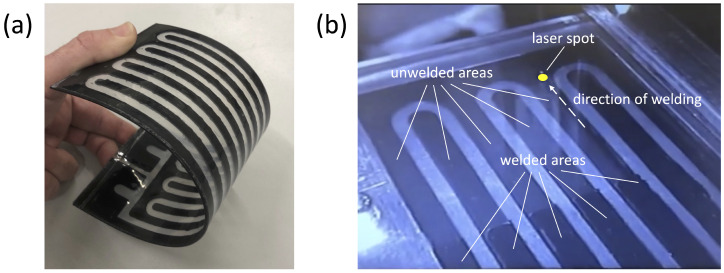
Flexible polypropylene pulsating heat pipe (**a**) and detail of the selective transmission laser welding process (**b**).

A limited number of attempts have been carried out to fabricate PHPs using polymer materials. Lin et al. [130] developed and tested a flat-plate transparent PDMS PHP with copper heat source and sink. It was found that the thermal performance is quite low in comparison with copper PHPs of equal size. A similar device was proposed by Ji et al. [131], but the PHP exhibited better thermal performance. Ogata et al. [132] built an ultra-thin transparent PHP using polyethylene terephthalate polymer for the PHP casing and UV-curable polymer resins for the PHP channel. The minimum thermal resistance was found to be almost equal to the thermal resistance of a copper plate having the same thickness of the PHP. Lim and Kim [133] manufactured several polymer pulsating heat pipes using a multilayer laminated film composed of aluminium and low-density polyethylene (LDPE) layers, with an indium coating applied on the PHP edges to prevent gas permeation into the PHP channel. The device exibited a good performance, achieving a minimum thermal resistance of 2.41 K/W. Jung et al. [134] introduced a pulsating heat pipe built with polycarbonate and sealed with polyimide and a copper casing. Arai and Kawaji [135] fabricated transparent polycarbonate PHPs by means of additive manufacturing. Very recently, Der and co-workers [136,137,138] developed a number of flat-plate fully polymeric PHPs having a meandering channel embedded in a composite polypropylene sheet (average thermal conductivity: 0.16 W/mK), characterised by high mechanical flexibility, as shown in Figure 8a. In particular, the composite polypropylene sheet is composed of three layers (250 mm × 100 mm) bonded by selective transmission laser welding, which is illustrated schematically in Figure 8b. The thermal performance of these devices was extensively characterized for several design and operation parameters, including different bending angles [101,139]. In all conditions, the PHPs achieve a minimum equivalent thermal resistance having a magnitude of about 2.3–4 K/W, i.e., one quarter to one third of the equivalent thermal resistance of the pulsating heat pipe envelope without heat transfer fluid (about 11 K/W).

## 4. Limitations of Polymer Materials

Although polymer materials represent an interesting and often desirable alternative to metals, they also have some peculiar properties (e.g., a relatively low thermal conductivity) that can make their use difficult in thermal management systems. In the followng sections, the main issues that jeopardize the use of polymer materials in thermal systems are discussed, along with solutions to mitigate their impact on the performance and/or the lifetime of two-phase passive heat transfer devices.

### 4.1. Thermal Conductivity

**Figure 9:**
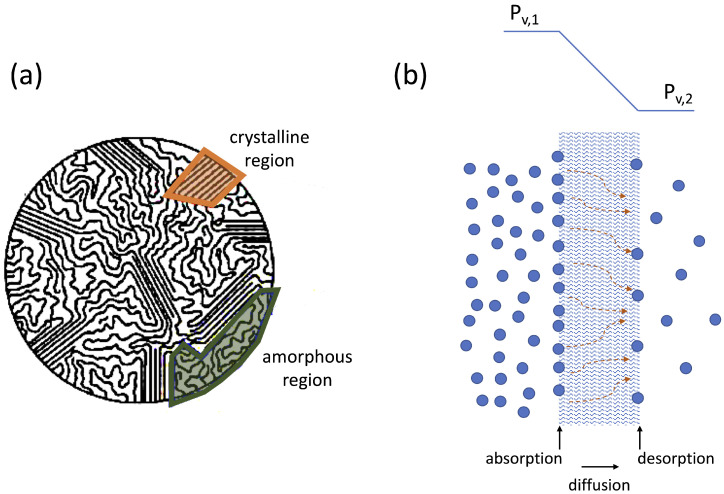
Sketch of a polymer morphology consisting of crystalline and amorphous regions (**a**) and schematic of the gas permeation process through a polymer separating two regions where a gas species has different partial pressures, $P_{v,1}>P_{v,2}$ (**b**).

Thermal conductivity measures the ability of a solid, liquid or gaseous medium to transfer heat by means of molecular diffusion, i.e., by random collisions at molecular level. Due to their high molecular weight and the large number of internal degrees of freedom of their molecules, polymer materials have low thermal conductivities, typically less than 1 W/m·K [140,141]. The morphology of polymers chains often consists of small portions of crystalline regions (where molecules are structured or systematically aligned), surrounded by amorphous regions (where molecules are randomly entangled), as shown in Figure 9a. In such amorphous regions, the thermal energy transferred by molecular diffusion is absorbed to activate the internal degrees of freedom of long polymer chains, significantly reducing the thermal conductivity as compared, e.g., with the crystalline microstructure of metals [1,13]. Thus, while they are widely used as insulators, they cannot be implemented whenever efficient heat transfer is expected.

One common method to improve the thermal conductivity of polymers is to realign polymer chains in order to increase the crystalline to amorphous ratio using either mechanical stretching (e.g., extrusion, lamination), nanoscale templating, or electrospinning [13]. However, increasing the crystallinity of polymers significantly affects their mechanical properties, reducing flexibility and resilience and increasing brittleness. Alternatively, one can significantly improve the thermal conductivity with a limited effect on mechanical properties by embedding a high thermal conductivity filler into the polymer matrix [142]. Such fillers include graphite, carbon black and fibres, ceramic or metal particles [143,144,145,146,147].

Carbon-based fillers, such as graphite, are characterized by high thermal conductivity, low weight, low cost and fair dispersability into a polymer matrix [148,149]. Expanded graphite, a type of exfoliated graphite composed of layers of 20–100 nm thickness, is also used in polymer composites [150]. Its effective thermal conductivity varies with the degree of exfoliation [151], the dispersion in matrix [152] and the aspect ratio of the filler [153]. An important limitation of this approach to enhance the thermal conductivity of polymers is it requires high filler loads (typically greater than 30% volume fractions). This represents a significant challenge for the material processing, in terms of both mouldability and welding, and severely limits their potential use in heat exchanger applications. Furthermore, highly loaded polymer composites typically present microstructural flaws (e.g., microvoids), due to inhomogeneous composition and to imperfections in the microparticles dispersion, which become a severe concern when moulding thin components is required and/or when the material permeability to volatile species is an issue.

A sensible target is to develop conductive polymer composites with thermal conductivity ≥10 W/m·K and containing relatively low amount of fillers (≤20% volume fraction). This could be achieved using graphene nanoplatelet fillers, which have a thermal conductivity of 800 W/m·K [154] (although its theoretical value can be as high as 5300 W/m·K [31]). In addition, the combination of micro- and nano-particles such as graphite and graphene can improve the efficiency of thermal exchange between conductive particles, thanks to the bridging effect of nanometric graphene layers between micrometric graphite particles.

### 4.2. Permeability

Unlike metals, polymers are permeable to certain non-condensable gas and vapor species due to both the intrinsic presence of molecular cavities in amorphous macromolecular entanglements, and to voids, cracks or other imperfections generated during material processing [155]. Permeation is a mass transfer process of gas or vapor molecules through a polymer material driven by a pressure difference between two surfaces, as shown in Figure 9b. This process occurs in three stages: (i) absorption of the gas/vapor into the polymer surface exposed to a higher pressure; (ii) diffusion of molecules through the polymer material; (iii) desorption of the gas/vapor from the polymer surface exposed to a lower pressure. Consequently, if the operating pressure of the heat transfer fluid is significantly different from the ambient pressure, which is the case with most two-phase heat transfer devices; polymer materials may not guarantee long-term gas tightness, which results in a progressive decay of the thermal performance.

Typically, a significant reduction or suppression of gas permeability can be achieved, for example, by applying surface coatings, by using laminated materials incorporating one or more impermeable layers, or by blending different materials [156,157,158]. Besides metallic coatings and barriers, thin films of Al_2_O_3_ grown by atomic layer deposition were shown to act effectively as gas a moisture barrier on a range of different polymer substrates [159,160,161]. Composite polymers loaded with lamellar nanoparticle fillers such as graphene also exhibit a reduced permeability since the filler creates tortuous pathways for molecules diffusing through the polymer matrix. In the case of pulsating heat pipes, this issue can be successfully resolved by applying suitable surface coatings or by embedding a thin metallic layer in the polymer acting as a gas barrier [81,133,134].

In the case of devices built with polymer or composite materials, the problem of gas/moisture permeation through the material is closely related to that of sealing, which arises wherever different components of a device are connected or bonded together. In fact, both problems result into a change of the operation pressure of the heat transfer fluid, which in turn affects the operation temperature and consequently the thermal performance of the device. Whilst sealing is a major issue in laboratory prototypes because of the usually large number of fittings and valves necessary to insert measurement probes and to enable frequent filling, emptying and/or vacuuming, it is less important in commercial devices, which are often enclosed into a single casing.

### 4.3. Wettability

**Figure 10:**
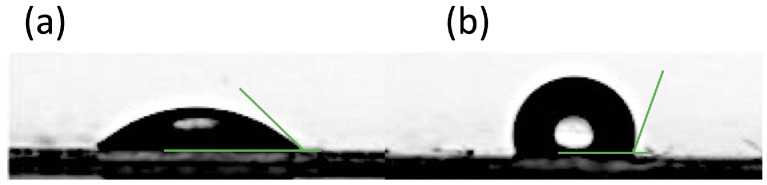
Contact angle of a water drop on (**a**) a polymer surface with medium surface energy ($\theta =44^{\circ })$ and (**b**) a polymer surface with low surface energy ($\theta =110^{\circ }$).

Surface wettability is the ability of a fluid to spread over a solid surface or to adhere to it, and is determined by the balance of intermolecular forces in the vicinity of the three-phase (liquid–solid–vapor) contact line, which is usually expressed in terms of interfacial tensions between the solid and the vapor, $\gamma_{SV}$, the solid and the liquid, $\gamma_{SL}$ and the liquid and the vapor, $\gamma_{LV}$, respectively. This results in the well-known Young’s equation, $\gamma_{SV}=\gamma_{SL}+\gamma_{LV}\cos\theta$ [162], where θ is the angle between the solid surface and the liquid–air interface on the side of the liquid phase, or the contact angle. Although Young’s equation was originally understood as the balance of three surface tensions, it can be derived more rigorously by interpreting $\gamma_{SV}$, $\gamma_{SL}$ and $\gamma_{LV}$ as interfacial energies per unit area, and minimizing the total free energy of the system [163,164]. According to Young’s equation, a liquid with a given surface tension, $\gamma_{LV}$, will tend to wet completely materials with high surface energy, $\gamma_{SV}$ (cosθ≈1), while on materials with low surface energy cosθ will have small or even negative values, resulting in larger contact angles [165]. The two cases of high and low surface energies (i.e., small and large contact angles) are displayed in Figure 10a,b, respectively.

While metals and most ceramics typically have large surface energies, therefore they are wetted by most liquids, the surface energy of polymer materials is significantly smaller, which makes their surface poorly wettable [166]. In composite materials, a suitable choice of the filler can also improve wettability [158].

Since wettability is directly responsible for capillary forces [164], it is a critical property in two-phase passive heat transfer devices where the heat transfer fluid condensate is driven by capillarity, such as heat pipes, capillary loops and pulsating heat pipes. In particular, if the heat transfer fluid does not wet the channel wall sufficiently, circulation must be assisted by gravity in order to obtain a good heat transfer performance [139].

To improve polymers surface wettability, it is necessary to modify the molecular structure of the surface using a range of surface treatment processes such as laser beam and plasma surface treatments [167,168], or coating the polymer surfaces in contact with the fluid with high surface energy materials such as metals.

### 4.4. Viscoelasticity

Although it is desirable in a number of applications (e.g., deployable systems and flexible or foldable electronics), the mechanical flexibility of polymer materials can have unwanted effects on the operation of heat transfer devices. In particular, the deformation of polymeric walls due, e.g., to variations of the ambient pressure affects the pressure of the heat transfer fluid, both because of the volume change, and because the pressure difference between the external and the internal pressure must balance the stress buildup in the deformed channel walls, which acts to recover the initial shape. More importantly, the channel wall deformation affects the hydraulic diameter; this is of particular relevance in the case of pulsating heat pipes, where a variation of the hydraulic diameter may cause the Bond number to fall outside of the optimal range, reducing the thermal performance of the device [137]. An additional complication to account for is that in polymeric materials the deformation stress is not constant, but is subject to stress relaxation, i.e., the stress decay in response to a constant strain in the material. Similarly, when a constant force is applied to the material, the deformation rate decays exponentially depending on the relaxation time of the material. Such viscoelastic behaviour is usually described using either the Maxwell or the Kelvin–Voigt constitutive equations, or a combination of them [15]. The characteristic time of the material response to an applied stress or deformation, or relaxation time, can range from a few seconds in elastomers to several hours or even days for certain polymers, and sometimes exceeds the operational thermal transients of a device built with such materials. Both the mechanical properties (Young’s and flexural moduli) and the relaxation time are strongly dependent on temperature, because increasing the material temperature activates a larger numbers of internal degrees of freedom and reptation modes of the polymer chains [169,170], which makes polymer materials softer and subjected to larger deformations in a shorter time for a given applied stress. On the opposite side, temperatures below the glass transition temperature range cause a sharp increase of the Young’s and flexural moduli as well as a sharp drop of the ultimate strength. As a consequence, the use of polymer materials in heat transfer devices is possible only if the operating temperature range falls between the glass transition point and the softening point of the material.

## 5. Conclusions

The use of polymer and composite materials to fabricate two-phase passive thermal management systems has been reviewed critically, with an assessment of their limitations and technical challenges. Although two-phase passive systems are often built with metallic materials because of their good mechanical and thermal properties, recent advances in technology such as the develoment of portable or deployable systems, including foldable and flexible electronics, are making the use of polymer and composite materials more and more attractive in order to meet technical requirements of mechanical flexibility and low weight, as well as cost-effectiveness. Consequently, several attempts to use these materials to replace metals (either partially or completely) in passive thermal management systems are documented in the literature.

Polymeric two-phase passive heat transfer devices and thermal management systems are generally efficient and exhibit relatively high thermal performances. However, due to the low thermal conductivity, the poor surface wettability, the high gas-vapour permeability, and the viscoelastic behaviour of most polymer materials, the performances are usually not comparable to those achieved using metals. In addition, the decay of the thermal performances in time significantly affects the lifetime of devices, despite these materials are not subject to corrosion. At present, the most promising route to overcome these limitations is the development of polymer-based composites.

## Figures and Tables

**Table 1:** Classification of the most common polymeric materials of commercial interest for the construction of heat transfer devices.

Thermoplastics	Thermosets	Elastomers
Polyethylene (PE)	Polyesters	Polyisoprene (natural rubber, isoprene rubber)
Low density polyethylene (LDPE)	Epoxy	Styrene-butadiene copolymer (styrene-butadiene rubber)
High density polyethylene (HDPE)	Melamine formaldehyde	Polybutadiene (butadiene rubber)
Polypropylene (PP)	Urea formaldehyde	Acrylonitrile-butadiene copolymer (nitrile rubber)
Poly(vinyl chloride) (PVC)	Polyurethane	Isobutylene-isoprene copolymer (butyl rubber)
Polystyrene (PS)	Phenol formaldehyde (PF)	Ethylene-propylene monomer (EPM), ethylene-propylene-diene monomer (EPDM)
Acrylonitrile butadiene styrene (ABS)	Silicone	Polychloroprene (neoprene)
Polycarbonate (PC)	Duroplast	Polysulfide (Thiokol)
Acrylic	Cyanate Ester	Polydimethyl siloxane (silicone)
Acrylonitrile butadiene styrene (ABS)	Polyimide	Fluoroelastomer
Nylon	Furan	Polyacrylate elastomer
Polylactic acid (polylactide)	Vinyl Ester	Polyethylene (chlorinated, chlorosulfonated)
Polyether sulfone (PES)	Vulcanized Rubber	Styrene-isoprene-styrene (SIS), styrene-butadiene-styrene (SBS) block copolymer
Polyoxymethylene (POM)	Bakelite	EPDM-polypropylene blend
Polyether ether ketone(PEEK)	Thiolyte	
Polytetrafluoroethylene (Teflon)	Benzoxazines	
Polyetherimide (PEI)	Diallyl-phthalate (DAP)	

## Data Availability

Not applicable.

## Abbreviations

The following abbreviations are used in this manuscript:

PE	Polyethylene
LDPE	Low density polyethylene
HDPE	High density polyethylene
PP	Polypropylene
PVC	Poly(vinylchloride)
PS	Polystyrene
ABS	Acrylonitrile butadiene styrene
PC	Polycarbonate
PTFE	Polytetrafluoroethylene
PES	Polyether sulfone
POM	Polyoxymethylene
PEEK	Polyether ether ketone
PF	Phenol formaldehyde
EPM	Ethylene-propylene monomer
EPDM	Ethylene-propylene-diene monomer
SIS	Styrene-isoprene-styrene
SBS	Styrene-butadiene-styrene
TS	Thermosyphon
HP	Heat pipe
LHP	Loop heat pipe
CPL	Capillary pumped loop
PHP	Pulsating heat pipe
OHP	Oscillating heat pipe
LED	Light-emitting diode
RF	Radio frequency
PCB	Printed circuit board
LCP	Liquid crystal polymer
LDPET	Low density polyethylene terephthalate
PET	Polyethylene terephthalate
UHMW-PE	Ultra-high-molecular-weight polyethylene
PDMS	Polydimethylsiloxane

## References

[1] Kim H.S., Jang J.U., Lee H., Kim S.Y., Kim S.H., Kim J., Jung Y.C., Yang B.J. (2018). Thermal Management in Polymer Composites: A Review of Physical and Structural Parameters. Adv. Eng. Mater..

[2] Zhang H., Shi T., Ma A. (2021). Recent Advances in Design and Preparation of Polymer-Based Thermal Management Material. Polymers.

[3] Wang B., Li G., Xu L., Liao J., Zhang X. (2020). Nanoporous boron nitride aerogel film and its smart composite with phase change materials. ACS Nano.

[4] Li W., Wang F., Cheng W., Chen X., Zhao Q. (2020). Study of using enhanced heat-transfer flexible phase change material film in thermal management of compact electronic device. Energy Convers. Manag..

[5] Li C., Li J. (2021). Passive Cooling Solutions for High Power Server CPUs with Pulsating Heat Pipe Technology. Front. Energy Res..

[6] Gibbons M.J., Marengo M., Persoons T. (2021). A review of heat pipe technology for foldable electronic devices. Appl. Therm. Eng..

[7] Ruch P., Brunschwiler T., Escher W., Paredes S., Michel B. (2011). Toward five-dimensional scaling: How density improves efficiency in future computers. IBM J. Res. Dev..

[8] Vasiliev L.L. (2005). Heat pipes in modern heat exchangers. Appl. Therm. Eng..

[9] Zohuri B. (2016). Heat Pipe Design and Technology: Modern Applications for Practical Thermal Management.

[10] Strobl G. (1997). The physics of polymers.

[11] Saldívar-Guerra E., Vivaldo-Lima E. (2013). Introduction to Polymers and Polymer Types. Handbook of Polymer Synthesis, Characterization, and Processing.

[12] Maqbool M., Aftab W., Bashir A., Usman A., Guo H., Bai S. (2022). Engineering of polymer-based materials for thermal management solutions. Compos. Commun..

[13] Huang C., Qian X., Yang R. (2018). Thermal conductivity of polymers and polymer nanocomposites. Mater. Sci. Eng. R Rep..

[14] Chen H., Ginzburg V.V., Yang J., Yang Y., Liu W., Huang Y., Du L., Chen B. (2016). Thermal conductivity of polymer-based composites: Fundamentals and applications. Prog. Polym. Sci..

[15] Lakes R., Lakes R. (2009). Viscoelastic Materials.

[16] Glade H., Moses D., Orth T., Bart H.J., Scholl S. (2018). Polymer Composite Heat Exchangers. Innovative Heat Exchangers.

[17] Stern S.A., Fried J.R., Mark J.E. (2007). Permeability of Polymers to Gases and Vapors. Physical Properties of Polymers Handbook.

[18] Harvey J.A., Kutz M. (2005). Chapter 7 - Chemical and physical aging of plastics. Handbook of Environmental Degradation of Materials.

[19] White J., De S.K. (2001). Rubber Technologist’s Handbook.

[20] Ghosh A.K., Dwivedi M. (2020). Advantages and applications of polymeric composites. Processability of Polymeric Composites.

[21] Liem H., Choy H. (2013). Superior thermal conductivity of polymer nanocomposites by using graphene and boron nitride as fillers. Solid State Commun..

[22] Xie B.H., Huang X., Zhang G.J. (2013). High thermal conductive polyvinyl alcohol composites with hexagonal boron nitride microplatelets as fillers. Compos. Sci. Technol..

[23] Mamunya Y.P., Davydenko V., Pissis P., Lebedev E. (2002). Electrical and thermal conductivity of polymers filled with metal powders. Eur. Polym. J..

[24] Krupa I., Novák I., Chodák I. (2004). Electrically and thermally conductive polyethylene/graphite composites and their mechanical properties. Synth. Met..

[25] Shahil K.M., Balandin A.A. (2012). Graphene-multilayer graphene nanocomposites as highly efficient thermal interface materials. Nano Lett..

[26] Zhang C., Liu T. (2012). A review on hybridization modification of graphene and its polymer nanocomposites. Chin. Sci. Bull..

[27] Han Z., Fina A. (2011). Thermal conductivity of carbon nanotubes and their polymer nanocomposites: A review. Prog. Polym. Sci..

[28] Wang M., Kang Q., Pan N. (2009). Thermal conductivity enhancement of carbon fiber composites. Appl. Therm. Eng..

[29] Videira-Quintela D., Martin O., Montalvo G. (2021). Recent advances in polymer-metallic composites for food packaging applications. Trends Food Sci. Technol..

[30] Yoo B.M., Shin H.J., Yoon H.W., Park H.B. (2014). Graphene and graphene oxide and their uses in barrier polymers. J. Appl. Polym. Sci..

[31] Stankovich S., Dikin D.A., Dommett G.H., Kohlhaas K.M., Zimney E.J., Stach E.A., Piner R.D., Nguyen S.T., Ruoff R.S. (2006). Graphene-based composite materials. Nature.

[32] Lizundia E., Vilas J.L., Sangroniz A., Etxeberria A. (2017). Light and gas barrier properties of PLLA/metallic nanoparticles composite films. Eur. Polym. J..

[33] Wen S., Zhang R., Xu Z., Zheng L., Liu L. (2020). Effect of the topology of carbon-based nanofillers on the filler networks and gas barrier properties of rubber composites. Materials.

[34] Su K.H., Su C., Cho C.T., Lin C.H., Jhou G.F., Chang C.C. (2019). Development of Thermally Conductive Polyurethane Composite by Low Filler Loading of Spherical BN/PMMA Composite Powder. Sci. Rep..

[35] Hill R.F., Supancic P.H. (2004). Determination of the thermal resistance of the polymer–ceramic interface of alumina-filled polymer composites. J. Am. Ceram. Soc..

[36] Lee W., Han I., Yu J., Kim S., Byun K. (2007). Thermal characterization of thermally conductive underfill for a flip-chip package using novel temperature sensing technique. Thermochim. Acta.

[37] Yu S., Hing P., Hu X. (2002). Thermal conductivity of polystyrene-aluminum nitride composite. Compos. Part A Appl. Sci. Manuf..

[38] Pezzotti G., Kamada I., Miki S. (2000). Thermal conductivity of AlN/polystyrene interpenetrating networks. J. Eur. Ceram. Soc..

[39] Morelli D., Heremans J. (2002). Thermal conductivity of germanium, silicon, and carbon nitrides. Appl. Phys. Lett..

[40] Azeem M., Jan R., Farrukh S., Hussain A. (2019). Improving gas barrier properties with boron nitride nanosheets in polymer-composites. Results Phys..

[41] Biron M., Biron M. (2018). Chapter 6—Thermoplastic Composites. Thermoplastics and Thermoplastic Composites.

[42] Moumen A.E., Tarfaoui M., Lafdi K. (2019). Additive manufacturing of polymer composites: Processing and modeling approaches. Compos. Part B Eng..

[43] Chohan J.S., Boparai K.S., Singh R., Hashmi M. (2020). Manufacturing techniques and applications of polymer matrix composites: A brief review. Adv. Mater. Process. Technol..

[44] Chi S. (1976). Heat Pipe Theory and Practice: A Sourcebook.

[45] Bejan A., Kraus A.D. (2003). Heat Transfer Handbook.

[46] Messaoud B., Aidoun Z., Eslami-nejad P., Blessent D. (2019). Ground-Coupled Natural Circulating Devices (Thermosiphons): A Review of Modeling, Experimental and Development Studies. Inventions.

[47] Jumeau J. (2010). Little history of water circulating heaters and storage heater. History of Technologies Linked to Heating.

[48] El-Genk M., Saber H. (1998). Heat transfer correlations for liquid film in the evaporator of enclosed, gravity-assisted thermosyphons. J. Heat Transf. (Trans. ASME).

[49] Lamaison N., Ong C.L., Marcinichen J.B., Thome J.R. (2017). Two-phase mini-thermosyphon electronics cooling: Dynamic modeling, experimental validation and application to 2U servers. Appl. Therm. Eng..

[50] Oliveira J., Tecchio C., Paiva K., Mantelli M., Gandolfi R., Ribeiro L. (2016). In-flight testing of loop thermosyphons for aircraft cooling. Appl. Therm. Eng..

[51] Nithyanandam K., Pitchumani R. (2013). Thermal energy storage with heat transfer augmentation using thermosyphons. Int. J. Heat Mass Transf..

[52] Ersöz M.A. (2016). Effects of different working fluid use on the energy and exergy performance for evacuated tube solar collector with thermosyphon heat pipe. Renew. Energy.

[53] Ziapour B.M., Khalili M.B. (2016). PVT type of the two-phase loop mini tube thermosyphon solar water heater. Energy Convers. Manag..

[54] Zhang P., Wang B., Wu W., Shi W., Li X. (2015). Heat recovery from Internet data centers for space heating based on an integrated air conditioner with thermosyphon. Renew. Energy.

[55] Matsubara K., Nakakura M., Bellan S., Maezawa K. (2019). Loop thermosiphon thermal collector for waste heat recovery power generation. Exp. Heat Transf..

[56] Zhang H., Shao S., Tian C., Zhang K. (2018). A review on thermosyphon and its integrated system with vapor compression for free cooling of data centers. Renew. Sustain. Energy Rev..

[57] Cao J., Zheng Z., Asim M., Hu M., Wang Q., Su Y., Pei G., Leung M.K. (2020). A review on independent and integrated/coupled two-phase loop thermosyphons. Appl. Energy.

[58] Jafari D., Franco A., Filippeschi S., Di Marco P. (2016). Two-phase closed thermosyphons: A review of studies and solar applications. Renew. Sustain. Energy Rev..

[59] Gernert N.J., Donovan K.G. (1994). Unfurlable Radiator for Lunar Base Heat Rejection. SAE Trans..

[60] Sukchana T., Pratinthong N. (2017). Effect of bending position on heat transfer performance of R-134a two-phase close loop thermosyphon with an adiabatic section using flexible hoses. Int. J. Heat Mass Transf..

[61] Sukchana T., Pratinthong N. (2016). A two-phase closed thermosyphon with an adiabatic section using a flexible hose and R-134a filling. Exp. Therm. Fluid Sci..

[62] Grakovich L.P., Rabetskii M.I., Vasiliev L.L., Leonid L., Vasiliev J., Bogdanovich S.P., Pesetskii S.S. (2014). Polymer flat loop thermosyphons. Heat Pipe Sci. Technol. Int. J..

[63] Vasiliev L., Vassiliev L. (2016). Heat Pipes and Nanotechnologies. Microscale and Nanoscale Heat Transfer: Analysis, Design, and Application.

[64] Chen B.R., Changa Y.W., Leeb W.S., Chen S.L. (2009). Long-term thermal performance of a two-phase thermosyphon solar water heater. Sol. Energy.

[65] Garrity P.T., Klausner J.F., Mei R. (2009). Instability phenomena in a two-phase microchannel thermosyphon. Int. J. Heat Mass Transf..

[66] Khodabandeh R., Furberg R. (2010). Instability, heat transfer and flow regime in a two-phase flow thermosyphon loop at different diameter evaporator channel. Appl. Therm. Eng..

[67] He Y., Hu C., Li H., Hu X., Tang D. (2022). Visualized-experimental investigation on a mini-diameter loop thermosyphon with a wide range of filling ratios. Int. Commun. Heat Mass Transf..

[68] Voirand A., Lips S., Sartre V. (2020). Heat transfer and flow visualizations in a flat confined two-phase thermosyphon. Int. J. Heat Mass Transf..

[69] Gaugler R.S. (1942). Heat Transfer Device. U.S. Patent.

[70] Grover G.M. (1966). Evaporation-Condensation Heat Transfer Device. U.S. Patent.

[71] Yadavalli Y., Weibel J.A., Garimella S.V. (2015). Performance-governing transport mechanisms for heat pipes at ultrathin form factors. IEEE Trans. Components Packag. Manuf. Technol..

[72] Feng C., Gibbons M., Marengo M., Chandra S. (2020). A novel ultra-large flat plate heat pipe manufactured by thermal spray. Appl. Therm. Eng..

[73] Tang H., Tang Y., Wan Z., Li J., Yuan W., Lu L., Li Y., Tang K. (2018). Review of applications and developments of ultra-thin micro heat pipes for electronic cooling. Appl. Energy.

[74] Chen X., Ye H., Fan X., Ren T., Zhang G. (2016). A review of small heat pipes for electronics. Appl. Therm. Eng..

[75] Ling L., Zhang Q., Yu Y., Liao S. (2021). A state-of-the-art review on the application of heat pipe system in data centers. Appl. Therm. Eng..

[76] Shewale S.P., Sahu S.K., Chougule S.S., Pise A.T. A review of heat pipe with nanofluid for electronic cooling. Proceedings of the 2014 International Conference on Advances in Engineering and Technology (ICAET).

[77] Wits W.W., Vaneker T.H. (2010). Integrated design and manufacturing of flat miniature heat pipes using printed circuit board technology. IEEE Trans. Components Packag. Technol..

[78] Oshman C., Shi B., Li C., Yang R., Lee Y., Peterson G., Bright V.M. (2011). The development of polymer-based flat heat pipes. J. Microelectromechanical Syst..

[79] Oshman C., Li Q., Liew L.A., Yang R., Lee Y.C., Bright V.M., Sharar D.J., Jankowski N.R., Morgan B.C. (2012). Thermal performance of a flat polymer heat pipe heat spreader under high acceleration. J. Micromechanics Microengineering.

[80] Shi B., Wang Y.B., Shan Y.J. (2016). An Experimental Investigation of Thermal Performance of a Polymer-Based Flat Heat Pipe. Heat Transf. Res..

[81] Oshman C., Li Q., Liew L.A., Yang R., Bright V.M., Lee Y. (2012). Flat flexible polymer heat pipes. J. Micromechanics Microengineering.

[82] Shih W.P., Wu G.W., Chen S.L. (2012). Lamination and Characterization of a Polyethylene-Terephthalate Flexible Micro Heat Pipe. Front. Heat Pipes.

[83] Savino R., di Francescantonio N., Fortezza R., Abe Y. (2007). Heat pipes with binary mixtures and inverse Marangoni effects for microgravity applications. Acta Astronaut..

[84] Hsieh S.S., Yang Y.R. (2013). Design, fabrication and performance tests for a polymer-based flexible flat heat pipe. Energy Convers. Manag..

[85] Lewis R., Liew L.A., Xu S., Lee Y.C., Yang R. (2015). Microfabricated ultra-thin all-polymer thermal ground planes. Sci. Bull..

[86] Yang K.S., Yang T.Y., Tu C.W., Yeh C.T., Lee M.T. (2015). A novel flat polymer heat pipe with thermal via for cooling electronic devices. Energy Convers. Manag..

[87] Yang C., Chang C., Song C., Shang W., Wu J., Tao P., Deng T. (2016). Fabrication and performance evaluation of flexible heat pipes for potential thermal control of foldable electronics. Appl. Therm. Eng..

[88] Yang C., Song C., Shang W., Tao P., Deng T. (2015). Flexible heat pipes with integrated bioinspired design. Prog. Nat. Sci. Mater. Int..

[89] Hou H., Xie Y., Li Q. (2005). Large-Scale Synthesis of Single-Crystalline Quasi-Aligned Submicrometer CuO Ribbons. Cryst. Growth Des..

[90] Huang J., Zhou W., Xiang J., Liu C., Gao Y., Li S., Ling W. (2020). Development of novel flexible heat pipe with multistage design inspired by structure of human spine. Appl. Therm. Eng..

[91] Butler D., Ku J., Swanson T. (2002). Loop heat pipes and capillary pumped loops-an applications perspective. Proceedings of the AIP Conference Proceedings.

[92] Riehl R.R., Dutra T. (2005). Development of an experimental loop heat pipe for application in future space missions. Appl. Therm. Eng..

[93] Zhao X., Wang Z., Tang Q. (2010). Theoretical investigation of the performance of a novel loop heat pipe solar water heating system for use in Beijing, China. Appl. Therm. Eng..

[94] Zhang X., Zhao X., Xu J., Yu X. (2013). Characterization of a solar photovoltaic/loop-heat-pipe heat pump water heating system. Appl. Energy.

[95] Vasiliev L., Lossouarn D., Romestant C., Alexandre A., Bertin Y., Piatsiushyk Y., Romanenkov V. (2009). Loop heat pipe for cooling of high-power electronic components. Int. J. Heat Mass Transf..

[96] Jose J., Baby R. (2018). Recent advances in loop heat pipes: A review. IOP Conf. Ser. Mater. Sci. Eng..

[97] Chen P.C., Lin W.K. (2001). The application of capillary pumped loop for cooling of electronic components. Appl. Therm. Eng..

[98] Gottschlich J.M. (1989). Capillary Pumped Loops for Aerospace Application. SAE Trans..

[99] Accorinti F., Ayel V., Bertin Y. (2019). Steady-state analysis of a Capillary Pumped Loop for Terrestrial Application with methanol and ethanol as working fluids. Int. J. Therm. Sci..

[100] Kobayashi T., Ogushi T., Haga S., Ozaki E., Fujii M. (2003). Heat transfer performance of a flexible looped heat pipe using R134a as a working fluid: Proposal for a method to predict the maximum heat transfer rate of FLHP. Heat Transf. Res..

[101] Alqahtani A.A., Edwardson S., Marengo M., Bertola V. (2022). Performance of flat-plate, flexible polymeric pulsating heat pipes at different bending angles. Appl. Therm. Eng..

[102] Wu S.C., Gu T.W., Wang D., Chen Y.M. (2015). Study of PTFE wick structure applied to loop heat pipe. Appl. Therm. Eng..

[103] Maydanik Y., Fershtater Y., Pastukhov V. (1989). Loop Heat Pipes: Development, Investigation and Elements of Engineering Calculations. Technical Report.

[104] Hoang T.T., O’Connell T.A., Ku J., Butler C.D., Swanson T.D. Miniature loop heat pipes for electronic cooling. Proceedings of the International Electronic Packaging Technical Conference and Exhibition.

[105] Gernert N.J., Brown J. (1995). Development of a Flexible Loop Heat Pipe Cold Plate. Proceedings of the Aerospace Atlantic Conference & Exposition.

[106] Ogushi T., Yao A., Xu J.J., Masumoto H., Kawaji M. (2003). Heat transport characteristics of flexible looped heat pipe under micro-gravity condition. Heat Transf. Res..

[107] Riehl R.R., Siqueira T.C. (2006). Heat transport capability and compensation chamber influence in loop heat pipes performance. Appl. Therm. Eng..

[108] Riehl R.R., Siqueira T. Evaluating loop heat pipes performances regarding their geometric characteristics. Proceedings of the International Conference on Environmental Systems-35th ICES.

[109] Adoni A.A., Ambirajan A., Jasvanth V., Kumar D., Dutta P. (2009). Effects of mass of charge on loop heat pipe operational characteristics. J. Thermophys. Heat Transf..

[110] Boo J.H., Chung W.B. (2005). Experimental study on the thermal performance of a small-scale loop heat pipe with polypropylene wick. J. Mech. Sci. Technol..

[111] Nagano H., Fukuyoshi F., Ogawa H., Nagai H. (2011). Development of an experimental small loop heat pipe with polytetrafluoroethylene wicks. J. Thermophys. Heat Transf..

[112] Kaya T., Hoang T.T. (1999). Mathematical modeling of loop heat pipes and experimental validation. J. Thermophys. Heat Transf..

[113] Mitomi M., Nagano H. (2014). Long-distance loop heat pipe for effective utilization of energy. Int. J. Heat Mass Transf..

[114] Kiper A.M. (1991). Investigation of Thermal-Fluid Mechanical Characteristics of The Capillary Pump Loop.

[115] Kolos K., Herold K., Kroliczek E., Swanson T. (1996). Flow visualization in capillary pumped loop systems. Proceedings of the AIP Conference Proceedings.

[116] Ye H., Sokolovskij R., van Zeijl H.W., Gielen A.W., Zhang G. (2014). A polymer based miniature loop heat pipe with silicon substrate and temperature sensors for high brightness light-emitting diodes. Microelectron. Reliab..

[117] Phan N., Nagano H. (2020). Fabrication and testing of a miniature flat evaporator loop heat pipe with polydimethylsiloxane and molding. Appl. Therm. Eng..

[118] Phan N., Nagano H. (2022). Novel hybrid structures to improve performance of miniature flat evaporator loop heat pipes for electronics cooling. Int. J. Heat Mass Transf..

[119] Chang X., Watanabe N., Nagai H., Nagano H. (2022). Visualization of thermo-fluid behavior of loop heat pipe with two evaporators and one condenser under various orientations with even heat loads. Int. J. Heat Mass Transf..

[120] Chang X., Watanabe N., Nagano H. (2019). Visualization study of a loop heat pipe with two evaporators and one condenser under gravity-assisted condition. Int. J. Heat Mass Transf..

[121] Wang X., Yang J., Wen Q., Shittu S., Liu G., Qiu Z., Zhao X., Wang Z. (2022). Visualization study of a flat confined loop heat pipe for electronic devices cooling. Appl. Energy.

[122] Liu L., Yuan B., Cui C., Yang X., Wei J. (2022). Investigation of a loop heat pipe to achieve high heat flux by incorporating flow boiling. Int. J. Heat Mass Transf..

[123] Akachi H. (1990). Structure of Heat Pipe. U.S. Patent.

[124] Nine M.J., Tanshen M.R., Munkhbayar B., Chung H., Jeong H. (2014). Analysis of pressure fluctuations to evaluate thermal performance of oscillating heat pipe. Energy.

[125] Yang H., Khandekar S., Groll M. (2008). Operational limit of closed loop pulsating heat pipes. Appl. Therm. Eng..

[126] Alhuyi Nazari M., Ahmadi M.H., Ghasempour R., Shafii M.B., Mahian O., Kalogirou S., Wongwises S. (2018). A review on pulsating heat pipes: From solar to cryogenic applications. Appl. Energy.

[127] Han X., Wang X., Zheng H., Xu X., Chen G. (2016). Review of the development of pulsating heat pipe for heat dissipation. Renew. Sustain. Energy Rev..

[128] Marengo M., Nikolayev V.S. (2018). Pulsating Heat Pipes: Experimental Analysis, Design and Applications. Encyclopedia of Two-Phase Heat Transfer and Flow IV.

[129] Mameli M., Besagni G., Bansal P.K., Markides C.N. (2022). Innovations in pulsating heat pipes: From origins to future perspectives. Appl. Therm. Eng..

[130] Lin Y.H., Kang S.W., Wu T.Y. (2009). Fabrication of polydimethylsiloxane (PDMS) pulsating heat pipe. Appl. Therm. Eng..

[131] Ji Y., Liu G., Ma H., Li G., Sun Y. (2013). An experimental investigation of heat transfer performance in a polydimethylsiloxane (PDMS) oscillating heat pipe. Appl. Therm. Eng..

[132] Ogata S., Sukegawa E., Kimura T. Performance evaluation of ultra-thin polymer pulsating heat pipes. Proceedings of the Fourteenth Intersociety Conference on Thermal and Thermomechanical Phenomena in Electronic Systems (ITherm).

[133] Lim J., Kim S.J. (2018). Fabrication and experimental evaluation of a polymer-based flexible pulsating heat pipe. Energy Convers. Manag..

[134] Jung C., Lim J., Kim S.J. (2020). Fabrication and evaluation of a high-performance flexible pulsating heat pipe hermetically sealed with metal. Int. J. Heat Mass Transf..

[135] Arai T., Kawaji M. (2021). Thermal performance and flow characteristics in additive manufactured polycarbonate pulsating heat pipes with Novec 7000. Appl. Therm. Eng..

[136] Der O., Marengo M., Bertola V. A low cost, flexible pulsating heat pipe technology. Proceedings of the Thermal and Fluids Engineering Summer Conference.

[137] Der O., Marengo M., Bertola V. (2019). Thermal Performance of Pulsating Heat Stripes Built With Plastic Materials. J. Heat Transf..

[138] Der O., Edwardson S., Marengo M., Bertola V. (2019). Engineered composite polymer sheets with enhanced thermal conductivity. IOP Conf. Ser. Mater. Sci. Eng..

[139] Der O., Alqahtani A.A., Marengo M., Bertola V. (2021). Characterization of polypropylene pulsating heat stripes: Effects of orientation, heat transfer fluid, and loop geometry. Appl. Therm. Eng..

[140] Chae H.G., Kumar S. (2008). Making Strong Fibers. Science.

[141] Tarannum F., Muthaiah R., Annam R.S., Gu T., Garg J. (2020). Effect of alignment on enhancement of thermal conductivity of polyethylene–graphene nanocomposites and comparison with effective medium theory. Nanomaterials.

[142] Sato K., Horibe H., Shirai T., Hotta Y., Nakano H., Nagai H., Mitsuishi K., Watari K. (2010). Thermally conductive composite films of hexagonal boron nitride and polyimide with affinity-enhanced interfaces. J. Mater. Chem..

[143] Pierson H. (1994). Handbook of Carbon, Graphite, Diamond and Fullerenes: Properties, Processing and Applications.

[144] Fischer J., Gogotsi Y., Presser V. (2013). Carbon Nanotubes: Structure and Properties. Carbon Nanomaterials.

[145] Donnet J.B. (1993). Carbon Black: Science and Technology.

[146] Kelly B. (1981). Physics of Graphite.

[147] Causin V., Marega C., Marigo A., Ferrara G., Ferraro A. (2006). Morphological and structural characterization of polypropylene/conductive graphite nanocomposites. Eur. Polym. J..

[148] Tu H., Ye L. (2009). Thermal conductive PS/graphite composites. Polym. Adv. Technol..

[149] Ganguli S., Roy A.K., Anderson D.P. (2008). Improved thermal conductivity for chemically functionalized exfoliated graphite/epoxy composites. Carbon.

[150] de Sousa D.E.S., Scuracchio C.H., de Oliveira Barra G.M., de Almeida Lucas A., Friedrich K., Breuer U. (2015). Chapter 7—Expanded graphite as a multifunctional filler for polymer nanocomposites. Multifunctionality of Polymer Composites.

[151] Mu Q., Feng S. (2007). Thermal conductivity of graphite/silicone rubber prepared by solution intercalation. Thermochim. Acta.

[152] Kalaitzidou K., Fukushima H., Drzal L.T. (2007). Multifunctional polypropylene composites produced by incorporation of exfoliated graphite nanoplatelets. Carbon.

[153] Liu Z., Guo Q., Shi J., Zhai G., Liu L. (2008). Graphite blocks with high thermal conductivity derived from natural graphite flake. Carbon.

[154] Veca L.M., Meziani M.J., Wang W., Wang X., Lu F., Zhang P., Lin Y., Fee R., Connell J.W., Sun Y.P. (2009). Carbon Nanosheets for Polymeric Nanocomposites with High Thermal Conductivity. Adv. Mater..

[155] Duncan B., Urquhart J., Roberts S. (2005). Review of Measurement and Modelling of Permeation and Diffusion in Polymers.

[156] Anukiruthika T., Sethupathy P., Wilson A., Kashampur K., Moses J.A., Anandharamakrishnan C. (2020). Multilayer packaging: Advances in preparation techniques and emerging food applications. Compr. Rev. Food Sci. Food Saf..

[157] Rauschendorfer J.E., Vana P. (2021). Increasing the Gas Barrier Properties of Polyethylene Foils by Coating with Poly(methyl acrylate)-Grafted Montmorillonite Nanosheets. Polymers.

[158] Uthaman A., Lal H.M., Li C., Xian G., Thomas S. (2021). Mechanical and Water Uptake Properties of Epoxy Nanocomposites with Surfactant-Modified Functionalized Multiwalled Carbon Nanotubes. Nanomaterials.

[159] Groner M.D., George S.M., McLean R.S., Carcia P.F. (2006). Gas diffusion barriers on polymers using *Al*_2_*O*_3_ atomic layer deposition. Appl. Phys. Lett..

[160] Dameron A.A., Davidson S.D., Burton B.B., Carcia P.F., McLean R.S., George S.M. (2008). Gas Diffusion Barriers on Polymers Using Multilayers Fabricated by *Al*_2_*O*_3_ and Rapid *SiO*_2_ Atomic Layer Deposition. J. Phys. Chem. C.

[161] Langereis E., Creatore M., Heil S.B.S., van de Sanden M.C.M., Kessels W.M.M. (2006). Plasma-assisted atomic layer deposition of *Al*_2_*O*_3_ moisture permeation barriers on polymers. Appl. Phys. Lett..

[162] Young T. (1805). An essay on the cohesion of fluids. Philos. Trans. R. Soc. Lond..

[163] Gibbs J. (1961). The Scientific Papers of J.W. Gibbs.

[164] Israelachvili J. (2011). Intramolecular and Surface Forces.

[165] Agrawal G., Negi Y., Pradhan S., Dash M., Samal S., Tanzi M.C., Farè S. (2017). Wettability and contact angle of polymeric biomaterials. Characterization of Polymeric Biomaterials.

[166] Mittal K.L. (1983). Physicochemical Aspects of Polymer Surfaces.

[167] Berczeli M., Weltsch Z. (2021). Enhanced Wetting and Adhesive Properties by Atmospheric Pressure Plasma Surface Treatment Methods and Investigation Processes on the Influencing Parameters on HIPS Polymer. Polymers.

[168] Seiler M., Gruben J., Knauft A., Barz A., Bliedtner J. (2020). Laser beam activation of polymer surfaces for selective chemical metallization. Procedia CIRP.

[169] de Gennes P.G. (1971). Reptation of a Polymer Chain in the Presence of Fixed Obstacles. J. Chem. Phys..

[170] Doi M., Edwards S.F. (1978). Dynamics of concentrated polymer systems. Part 1.—Brownian motion in the equilibrium state. J. Chem. Soc. Faraday Trans..

